# Virally Suppressed People Living with HIV Who Use Opioids Have Diminished Latency Reversal

**DOI:** 10.3390/v15020415

**Published:** 2023-02-01

**Authors:** Binita Basukala, Sarah Rossi, Sally Bendiks, Natalia Gnatienko, Gregory Patts, Evgeny Krupitsky, Dmitry Lioznov, Kaku So-Armah, Manish Sagar, Christine Cheng, Andrew J. Henderson

**Affiliations:** 1 Department of Biology, Boston University, Boston, MA 02215, USA; 2 Clinical Addiction Research and Education (CARE) Unit, Department of Medicine, Section of General Internal Medicine, Boston Medical Center, Boston University Chobanian & Avedisian School of Medicine, Boston, MA 02118, USA; 3 Biostatistics and Epidemiology Data Analytics Center, Boston University School of Public Health, Boston, MA 02118, USA; 4 Laboratory of Clinical Pharmacology of Addictions, Pavlov First St. Petersburg State Medical University, Saint-Petersburg 197022, Russia; 5 Department of Addictions, Bekhterev National Medical Research Center for Psychiatry and Neurology, Saint-Petersburg 192019, Russia; 6 Department of Medicine, Section of Infectious Diseases, Boston University Chobanian & Avedisian School of Medicine, Boston, MA 02118, USA; 7 Department of Microbiology, Boston University Chobanian & Avedisian School of Medicine, Boston, MA 02118, USA; 8 Department of Psychiatry, University of California San Diego, La Jolla, CA 92093, USA

**Keywords:** opioid use, HIV pathogenesis, HIV latency

## Abstract

Of the 12 million people who inject drugs worldwide, 13% live with HIV. Whether opioid use impacts HIV pathogenesis and latency is an outstanding question. To gain insight into whether opioid use influences the proviral landscape and latent HIV reservoir, we performed intact proviral DNA assays (IPDA) on peripheral blood mononuclear cells (PBMCs) from antiretroviral therapy (ART)-suppressed people living with HIV (PWH) with or without current opioid use. No differences were observed between PWH with and without opioid use in the frequency of HIV intact and defective proviral genomes. To evaluate the latent reservoir, we activated PBMCs from ART-suppressed PWH with or without opioid use and assessed the induction of HIV RNA. PWH using opioids had diminished responses to ex vivo HIV reactivation, suggesting a smaller reversible reservoir of HIV-1 latently infected cells. However, in vitro studies using primary CD4+ T cells treated with morphine showed no effect of opioids on HIV-1 infection, replication or latency establishment. The discrepancy in our results from in vitro and clinical samples suggests that while opioids may not directly impact HIV replication, latency and reactivation in CD4+ T cells, opioid use may indirectly shape the HIV reservoir in vivo by modulating general immune functions.

## 1. Introduction

Opioids are a class of highly addictive drugs that are prescribed by medical practitioners for pain management and include heroin, morphine and fentanyl. Their addictive properties stem from the ability of opioids to activate reward circuits found within the mesocorticolimbic (mid-brain) dopamine system which results in a release of dopamine in the nucleus accumbens causing euphoria and reinforcement of drug-seeking behavior [1,2]. The opioid epidemic is a large burden on global health. According to a 2020 National Survey on Drug Use and Health, 9.5 million people misused opioids per year in the U.S., and 75% of the 92,000 drug overdose deaths are attributed to opioids [3,4]. Furthermore, opioid use is associated with increased risk of numerous infectious diseases, including HIV [5,6,7,8]. Approximately 10% of new HIV infections reported in 2021 are acquired due to injection drug use, with several risk factors such as shared needle use and unprotected sexual intercourse contributing to the enhancement of infection [9]. Some opioids, like morphine, methadone, buprenorphine, heroin and fentanyl, are also speculated to have immunomodulatory properties. Evidence for opioids directly influencing immune functions is based on in vitro cell models or animal studies. Several in vitro studies have reported immunosuppressive effects of opioids on multiple immune cell types, including suppression of macrophage phagocytosis and cytokine secretion, modulation of cytokine and chemokine production by neutrophils, dampening of cytotoxic function of natural killer cells, repression of dendritic cell functions and diminished B and T cell activation and proliferation [7,10]. 

In the context of HIV/AIDS, several studies have reported an association of opioid use with higher viral loads and faster progression to AIDS [8,11,12]. However, the role opioids play in HIV progression is confounded by people with opioid use disorders being less adherent to antiretroviral therapy (ART) [13,14,15]. In vitro experiments have also implicated opioids in regulating HIV infection and replication in human macrophages, peripheral blood mononuclear cells and lymphocytes [16,17]. In vitro treatment of these cells with opioids, mainly morphine, augments HIV replication by increasing the expression of HIV-1 co-receptors CCR5 and CXCR4 [18,19]. However, other studies report negative or no effects of opioid treatment on HIV replication in vitro, leading to conflicting opinions on the impact of opioids on HIV replication [20]. 

HIV infection results in the establishment of a pool of persistently infected, quiescent cells, such as resting and memory CD4+ T cell subsets, that harbor transcriptionally repressed latent replication-competent HIV-1 provirus [21,22,23]. This latent reservoir is long-lived with an estimated half-life of 44 months and is maintained by homeostatic proliferation, immune selection and clonal expansion [21,24,25,26,27,28]. The reservoir is also resistant to ART and fuels rapid viral rebound upon treatment in people with HIV (PWH), making HIV latency a major barrier to a cure [29]. The establishment of the latent reservoir is influenced by several cellular and molecular events, including the availability of transcription factors such as NF-κB, AP1 and NFAT which are essential for efficient proviral transcription, epigenetic regulation and RNA polymerase II processivity [30]. Immune activation and inflammation also shape the latent reservoir, with greater immune activation correlating with a larger reservoir size [31].

Opioids potentially influence the latent reservoir both directly and indirectly. Previous in vitro studies suggest that opioids suppress T cell activation as well as the activity of transcription factors including NF-κB, AP1, NFAT and CREB which regulate HIV transcription, [32,33]. Similarly, intravenous drug use, including opioids, is associated with increased inflammation and higher microbial translocation, exacerbating immune activation, which may influence latent reservoir establishment and maintenance [34,35,36]. The impact of opioids on the reservoir was suggested using an SIV-infected rhesus macaque model where morphine exposure was associated with a reduced latent reservoir in lymph nodes compared to control animals [35].

However, whether opioid usage impacts HIV latency in humans is not clear as there are few studies investigating the consequence of chronic opioid use on the latent reservoir in PWH. We hypothesize that opioid use influences the establishment and maintenance of the HIV reservoir in PWH. In this study, we aimed to investigate the effects of opioid use on the HIV latent reservoir by analyzing peripheral blood mononuclear cells (PBMCs) isolated from ART-suppressed PWH who reported recent opioid use at the time of enrollment compared to ART-suppressed PWH who reported no opioid use at enrollment. We did not observe differences in the presence of intact and defective proviral genomes that correlated with opioid use; however, we did observe that HIV reactivation was suppressed in PBMCs from PWH that were using opioids compared to control PWH not currently using opioids. Additionally, plasma TNF-α levels trended lower in PWH who used opioids compared to those without opioid use. In contrast to our results from clinical samples, we did not see any effects of direct morphine exposure on HIV-1 infection or latency reactivation in primary CD4+ T cells in vitro. These results provide insights into the role of opioid use and the impact on HIV latency in PWH.

## 2. Materials and Methods

### 2.1. Study Design

Samples used in this study were collected from participants enrolled in two randomized controlled trials: Linking Infection and Narcology Care Part II (LINC-II; R01DA045547) and Studying Partial Agonists for Ethanol and Tobacco Elimination in Russians with HIV (St PETER HIV ARCH U01AA020780). Both studies enrolled HIV-positive participants in St. Petersburg, Russia. LINC-II recruited hospitalized participants from a narcology hospital. All participants had a diagnosis of an opioid use disorder defined by DSM-5 [36], had a history of injection polydrug use that included heroin and methadone and were not on ART at study enrollment. Participants in the intervention arm of LINC-II received rapid access to ART, receipt of naltrexone for opioid use and 12 months of strength-based peer-led case management. St PETER HIV participants were heavy drinkers and daily smokers at enrollment. Participants were randomized to receive either varenicline, cytisine or nicotine replacement therapy with treatment regimens varying from 25 days to 12 weeks. Of relevance to this study, St. PETER HIV participants did not use opioids in the past 6 months, did not have an opioid use disorder diagnosis and had achieved viral suppression on ART. 

Thirty-five samples were from participants of the Investigating and Measuring Progression to AIDS in a Cohort Trial (IMPACT). IMPACT enrolled PWH who were ART naïve and heavy alcohol users. These samples were used for the validation of primers and probes for HIV-1 subtype A. 

Samples were collected from LINC-II intervention arm participants (*n* = 8) at baseline and at one follow-up visit, either at 6 or 12 months post-baseline, if participants achieved viral suppression. This study was performed using samples collected from LINC-II participants at the follow-up visit after they achieved viral suppression. Similarly, participants from St PETER HIV (*n* = 11) were enrolled into the current study at their final 12-month study visit if they met the criteria of being able to provide 50 mL of blood, had not used opioids in the past 6 months and did not have opioid use disorder. 

It should be noted that the study was initiated and samples were received from LINC-II and St PETER HIV studies before the 2022 Russia–Ukraine conflict and that no U.S. federal funds have been transferred to support this project in St. Petersburg since 2020. 

### 2.2. PBMC and CD4+ T Cell Isolation for In Vitro Experiments

Peripheral blood mononuclear cells were purified from de-identified blood leukapheresis packs by centrifugation using Lymphoprep gradient (StemCell Technologies, Vancouver, BC, Canada). CD4+ T cells were isolated by negative selection using EasySep Human CD4+ T Cell Enrichment Kits (StemCell Technologies, Vancouver, BC, Canada)). CD4+ T cells were maintained in RPMI-1640 supplemented with 10% FBS, 100 units/mL penicillin, 100 μg/mL streptomycin and 2 mM L-glutamine at 37 °C with 5% CO_2_. For CD4+ T cell activation, anti-CD3/CD28 beads (ThermoFisher Scientific, Waltham, MA, USA) were added to cells at the ratio of 1:1 cell:bead and incubated with IL-2 at 37 °C for 3 days. 

### 2.3. Packaging of Viruses for In Vitro Infections

HIV-1_NL4-3_ and HIV-1_Q23.17_ (subtype A) were used for infection of primary cells and generated by transfecting HEK293T cells with pNL4-3 and pQ23.17 using PEI transfection reagent (plasmids obtained from AIDS reagents program, Bethesda, MD, USA). Viral stocks of HIV-1_BRU-ΔEnv-GFP_ used for infection of primary CD4+ T cells were generated by co-transfecting HEK293T cells with pBRU-ΔEnv-GFP and pVSV-G (from AIDS reagents program). Viral supernatants were collected from the transfected cells and concentrated by ultracentrifugation over a sucrose gradient before being used to infect cells. Concentrated stocks were titrated in CEM-GFP cells engineered to express human CCR5 (AIDS reagents program). 

### 2.4. In Vitro HIV Infections

Morphine sulfate (Sigma Aldrich, Natick, MA, USA) was obtained with approval and oversight from the controlled substance sub-office of the Boston University Department of Environmental Health and Safety. Activated CD4+ T cells were treated with concentrations of morphine ranging from 10 nM to 100 mM for 24 h and infected by spinoculation with concentrated single-round BRU-ΔEnv-GFP virus, replication-competent NL4-3 or Q23.17 at 1200× *g* for 90 min. Cells were washed and cultured in complete RPMI-1640 in the presence of IL-2. Control wells were supplemented with 1 μM of the reverse transcriptase inhibitor Efavirenz (EFV). Cells were incubated for 3 days. Replication-competent NL4-3 infected cells were cultured for two weeks, and cells and culture supernatant were collected every 3 days. At the time of collection, cells were treated with DNase and MgCl_2_ to eliminate contaminating plasmid DNA. 

### 2.5. Quantitative Real-Time Alu-PCR

Total cell-associated DNA was extracted from cells using an AllPrep DNA/RNA mini kit (Qiagen, Germantown, MD, USA) according to the protocol. Integrated HIV-1 DNA was measured using qPCR as previously described [37]. PCR amplification of albumin was used as a standard to normalize gene copy number and genome number. For the quantification of integrated HIV-1 DNA, a nested Alu-gag PCR was performed as described previously [38,39]. Briefly, the first reaction was performed using forward primers for human Alu and reverse primers for HIV-1 Gag (Table 1). As a control for unintegrated HIV-1 DNA, a parallel reaction was run that contained only Gag primers. The first PCR reaction was performed on a TProfessional Thermocycler with the following program: hot start for 4 min at 95 °C and 20 cycles of 15 s at 93 °C, 15 s at 50 °C and 4 min at 70 °C. A second reaction was performed on the first reaction products with primer and probes for the HIV-1 R-U5 region (Table 1). 

### 2.6. RT-qPCR for Measuring RNA

Total cell-associated RNA from PBMCs and CD4+ T cells was extracted from cells using AllPrep DNA/RNA mini kit (Qiagen) according to the protocol. Forty milligrams of brain tissue derived from the inferior temporal gyrus (ITG) was used to isolate RNA with an RNeasy Plus Mini Kit (Qiagen). Five hundred nanograms of brain-derived RNA was utilized to generate a complementary DNA (cDNA) library using random primers (Promega, Madison, WI, USA). The RNA was prepared from tissues obtained from the Banner Sun Health Research Institute Brain and Body Donation Program (Sun City, Arizona) which is supported by the National Institute of Neurological Disorders and Stroke (U24 NS072026), the National Institute on Aging (P30 AG19610), the Arizona Department of Health Services (Contract 211002), the Arizona Biomedical Research Commission (Contracts 4001, 0011, 05-901 and 1001 to the Arizona Parkinson’s Disease Consortium) and the Michael J. Fox Foundation for Parkinson’s Research. A detailed protocol for cDNA library synthesis has been described previously [38,39]. Two microliters of cDNA from each sample was used for qPCR. The master mix consisted of the following: 7.5 μL GoTaq master mixture (Promega), 1.25 μL each forward and reverse primers prediluted at 5 μM (Invitrogen, Carlsbad, CA, USA). Primer sets used for the amplification of mu-opioid receptor, delta-opioid receptor, kappa opioid receptor, HIV-1 mRNA and RPL13a are detailed in Table 2. The reaction was run on QuantStudio 3 with the following settings: 15 min at 94 °C, followed by 45 cycles of 15 s at 94 °C, 30 s at 60 °C and 30 s at 72 °C. The relative level of HIV-1 mRNA was calculated using the ΔΔCt method. 

### 2.7. HIV p24 ELISA

A 96-well plate was coated with anti-HIV immunoglobin antibody (AIDS reagent program; catalog number 3957), and cell culture supernatants diluted with PBS with 10% FBS and 0.5% Triton-X were added to each well. Primary HIV p24 antibody (AIDS Reagent Program catalog number 183-H12-5C) was added to each well followed by the addition of HRP-coupled anti-mouse IgG antibody (Santa Cruz, Dallas, TX, USA). TMB (tetramethylbenzidine) peroxidase substrate (KPL) was added to the wells, and the color change of TMB substrate was measured in a TECAN Microplate reader at 450 nm. Quantification of p24 levels in the culture supernatant was performed against a commercially available p24 standard (Perkin Elmer, Waltham, MA, USA). 

### 2.8. Generation of Latently Infected Cells and Reactivation

Latently infected cells were generated in vitro as previously described [40]. Briefly, isolated naive CD4+ T cells from a healthy donor were activated with anti-CD3/CD28 beads (ThermoFisher Scientific, Waltham, MA, USA) added at the ratio of 1:1 (cell:bead) in the presence of anti-human IL-4 antibody (2 µg/10^6^ cells), anti-human IL-12 antibody (4 µg/10^6^ cells) and TGF-β1 (0.8 µg/10^6^ cells) (Peprotech, Rocky Hill, NJ, USA). The cells were maintained in RPMI medium supplemented with 10% FBS, 1% penicillin/streptomycin and 1% l-glutamine. After 3 days, magnetic anti-CD3/CD28 beads and TGF-β1 were removed and cells were resuspended in complete RPMI media supplemented with IL-2 (30 IU/mL) at a density of 10^6^ cells/mL. Media supplemented with IL-2 were replaced on days 4 and 5. To generate latently infected cells, 1/5th of the cultured T_CM_ cells were infected on day 7 with HIV_NL4-3_ by spinoculation at 1200× *g* for 90 min at room temperature at an MOI of 0.5. After infection, cells were mixed with 3/5th of the remaining T_CM_ cells and cultured at a density of 10^6^ cells/mL in complete RPMI medium with IL-2. On day 10, media and IL-2 were replaced, and the cells were cultured in 96-well round plates at a high density of 1 × 10^6^ cells per mL with 100 μL per well to ensure cell-to-cell transmission. On day 13, cells were transferred to flasks, media and IL-2 were replaced, and 100 nM of AMD-3100 or 1 μM of Raltegravir and 0.5 μM of Saquinavir were added to limit virus replication. On day 17, latently infected CD4+ T cells were enriched using Dynabeads CD4 Positive Isolation Kit (StemCell Technologies, Vancouver, Canada), and the percentage of p24+ cells was quantified by flow cytometry. 

For viral reactivation, cells were activated in presence of anti-CD3/CD28 beads at the ratio of 1 bead:1 cell for 48 h before harvesting for FACS and DNA/RNA isolation. 

### 2.9. Flow Cytometry

To monitor HIV-1 Gag expression, infected CD4+ T cells were fixed with BD CytoFix/CytoPerm buffer (BD Biosciences, Woburn, MA, USA) for 20 min and washed with Perm/Wash buffer (BD Biosciences). Cells were stained with anti-HIV-1 Gag antibody diluted in BD Perm/Wash and were then washed with BD Perm/Wash buffer, resuspended in fluorescence-activated cell sorting (FACS) buffer and analyzed using a BD LSRII flow cytometer. 

### 2.10. Intact Proviral DNA Assay

An intact proviral DNA assay (IPDA) was performed on DNA isolated from PBMCs from PWH as described previously [41]. Briefly, DNA was isolated from PBMCs using Qiagen AllPrep DNA/RNA mini kit to prevent excessive shearing of DNA. Primers and probes specific for the 5′ Psi and 3′ RRE region of the Env region were designed for subtype A HIV-1 by analyzing subtype A sequences deposited in the Los Alamos National Laboratory HIV database. We did not genotype HIV-1 sequences in the clinical samples used in this study. Each of these two regions was amplified by using a pair of forward and reverse primers that flank the probe sequence. The probe for 5′ Psi contained a FAM-labeled hydrolysis probe, while the 3′ RRE probe was a VIC-labeled hydrolysis probe. For correction of DNA shearing that could lead to overestimation of 5′ and 3′ deleted provirus and underestimation of intact provirus, a parallel ddPCR analysis for a host gene, RPP30, was performed on each sample as previously described [41]. DNA shearing and input cell numbers were calculated and applied for correction of experimentally observed DNA shearing. Primers and probes are shown in Table 3.

ddPCR Supermix for probes (no dUTPs) (Bio-Rad, Hercules, CA, USA) was used for the preparation of a master mix as described previously [41]. Droplets were generated using an automated droplet generator from BioRad, and ddPCR was performed on a BioRad QX200 droplet reader machine using manufacturer-approved consumables. QuantaSoft data analysis software was used for ddPCR data analysis (BioRad, Hercules, CA, USA). The number of cells assayed per sample varied and ranged from 12,321 to 529,112.

To ensure specificity, we compared the homology of the sequences for the Psi and Env specific primer and probe with the Psi and Env region of subtype A consensus sequence derived from the LANL HIV database. In the process of primer design, we ensured that we are amplifying the same region in subtype A as that of subtype B in the paper describing IPDA. In the LANL database, subtype A is further subdivided into sub-subtypes A1-A4 and A6. We designed primer and probe sequences to have 100% sequence homology to consensus sequences for all 5 sub-subtypes of subtype A. The primers and probes were validated by performing ddPCR on DNA isolated from PBMCs infected in vitro with an infectious clone of Q23.17, a subtype A virus. We were able to detect the presence of intact as well as psi-defective and env-defective provirus demonstrating that the primer and probe design was successful in detecting subtype A HIV-1 provirus in clinical samples (Supplementary Figure S1). 

### 2.11. Ex Vivo Activation of HIV Transcription from Clinical Samples

Freshly thawed PBMCs from PWH were counted. Half of the PBMCs were cultured in complete RPMI media alone while the remaining PBMCs were activated by incubation in the presence of anti-CD3/CD28 beads added at the ratio of 1 bead:1 cell for 3 days. To prevent the spread of infection, 1 μM of Efavirenz (EFV) and 1 μM of Saquinavir (SQV) were also added. Activated as well as unstimulated PBMCs were collected at the same time, and RNA isolation was performed using a Qiagen AllPrep DNA/RNA mini kit. 

### 2.12. Measuring HIV Transcription with RT-ddPCR Assay

For RT-ddPCR, cDNA was synthesized from 500 ng of RNA and a 20 μL reaction mixture with the following components: 4 μL of 5× 1st strand buffer (Invitrogen, Waltham, MA, USA), 2 μL of 0.1 M DTT (Invitrogen, Waltham, MA, USA), 2 μL of random hexamers (50 μg/mL; Promega, Madison, WI, USA), 1 μL of 10 mM deoxynucleoside triphosphates (dNTPs) mix, 0.1 μL of RNaseOut (40 U/μL; Invitrogen), 0.5 μL of SuperScript II RT (200 U/μL; Invitrogen, Waltham, MA, USA) and nuclease-free water to bring the final reaction volume to 20 μL. The reverse transcription reaction was carried out at 42 °C for 50 min followed by at 70 °C for 15 min. Negative controls containing no SuperScript II RT were also included in the reaction [42]. RT-ddPCR was performed using Bio-Rad QX200 AutoDG Digital Droplet PCR system. Primer sets used for the amplification of HIV-1 mRNA are detailed in Table 4. 

### 2.13. Plasma Cytokine Measurement

Frozen plasma samples were thawed and plasma levels of interleukin-6 (IL-6), interleukin-8 (IL-8) and tumor necrosis factor-alpha (TNF-α) were measured using bead-based LEGENDplex multi-analyte flow assay kit according to the manufacturer’s protocol (BioLegend, San Diego, CA, USA). Data were analyzed using LEGENDplex software version 8.0. 

### 2.14. Statistical Analysis

All statistical analyses were conducted using GraphPad Prism 9. To determine whether the differences between two groups were significant, Mann–Whitney U test was used to compare IPDA results and inflammatory cytokine levels between PWH with and without opioid use and correlation as determined by Spearman analysis. Statistical significance for in vitro experiments was analyzed using a *t*-test. Two-tailed α level was assumed for all statistical tests performed, and a *p* value of <0.05 was considered to be significant. Multiple variable logistic regression was used to assess odds for ex vivo reactivation. In this model, ex vivo reactivation was categorized as a categorical variable based on whether HIV-1 RNA transcripts were higher or lower with activation as compared to baseline. Years of ART, intact provirus per 10^6^ PBMCs and current opioid use were the predictors of interest.

### 2.15. Study Approval

The two randomized controlled trials, Linking Infection and Narcology Care Part II (LINC-II; R01DA045547) and Studying Partial Agonists for Ethanol and Tobacco Elimination in Russians with HIV (St PETER HIV ARCH U01AA020780), were approved by the institutional review boards of Boston University Medical Campus and First St. Petersburg Pavlov State Medical University. All LINC-II and St. PETER HIV ARCH participants provided written informed consent prior to participation in the current study. IMPACT participants provided consent for their stored biospecimens and data to be used for future HIV research. 

## 3. Results

### 3.1. HIV-1 Proviruses Are Comparable between PWH Currently Using Opioids and Not Using Opioids

The current HIV epidemic in Russia is driven by injection drug use, providing an opportunity to examine associations between opioid use, HIV pathogenesis and disease progression. In this study, we analyzed samples from a total of 19 PWH enrolled in two studies based in St. Petersburg, Russia (See Methods, Table 1). Eight samples were from participants in LINC-II who met the following criteria: reported using opioids in the past 30 days at the time of enrollment, a diagnosis of opioid use disorder (OUD), history of injection drug use and viral suppression on ART. Eleven participants from St PETER HIV were included in the current study if they met the following criteria: did not use opioids in the past 6 months, did not have an OUD diagnosis and had achieved viral suppression on ART (Table 5). 

**Figure 1:**
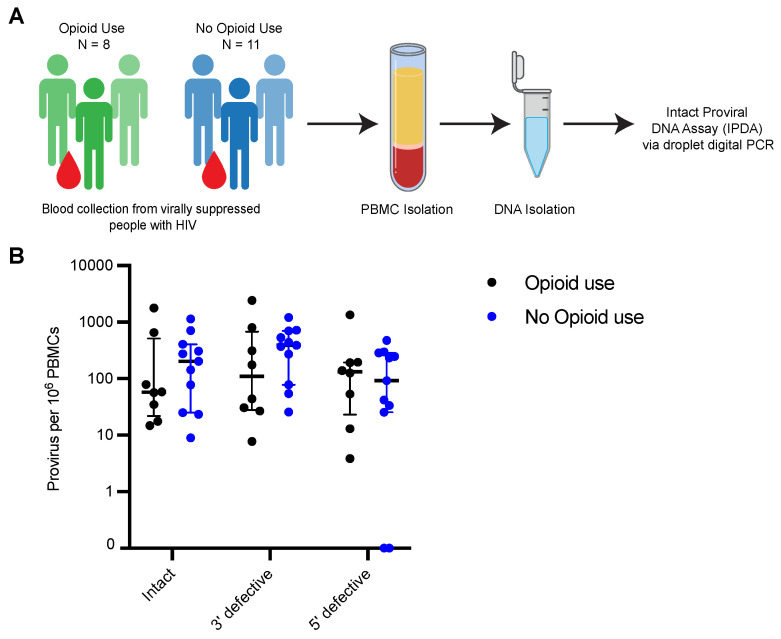
HIV-1 proviral landscape is comparable between people with HIV that currently use opioids and people with HIV not currently using opioids. (**A**) Experimental workflow for IPDA (**B**) IPDA results for intact proviruses, proviruses with 3’ defects and proviruses with 5’ defects in PBMCs from ART treated PWH with (*n* = 8) or without current opioid use (*n* = 11). Values are expressed as proviruses per 10^6^ PBMCs. Black and blue circles represent PWH using or not using opioids, respectively. Bars represent median with interquartile range. For (**B**), cells assayed per sample ranged from 12,321 to 529,216. A separate Rpp30 assay was performed to correct for DNA shearing and estimation of number of cells assayed per reaction.

Opioids have been suggested to influence T cell activation, function and HIV replication. Therefore, we were interested in assessing whether opioid use is associated with changes in the proviral latent reservoir in ART-suppressed PWH. To address this, we performed intact proviral DNA assays (IPDA) on DNA isolated from PBMCs from virally suppressed PWH without and with current opioid use (Figure 1A). IPDA differentiates between and quantifies intact and defective provirus present in infected cells [41]. Defective proviruses are characterized by the presence of large deletions and hypermutations which may affect the fitness of viruses, whereas intact proviruses are characterized by a lack of hypermutations and/or deletions and the ability to produce infectious particles [43,44]. IPDA was originally designed for the HIV-1 subtype B strain, which is the most prevalent strain in North America. However, in Russia, the most dominant strain is subtype A [45,46,47]. So, we designed primers and probes specific for the HIV-1 subtype A Psi and Env region which detected intact proviral sequences in CD4+ T cells infected in vitro with subtype A variant Q23.17 as well as clinical samples from PWH not receiving ART treatment (Supplementary Figure S1, Supplementary Table S1). A separate ddPCR assay was also performed for a cellular gene, *RPP30*, for quantitation of input cell number and DNA shearing correction which was used to correct IPDA values and normalize proviruses to the number of PBMCs [36].

For all samples, the median frequencies of intact, 3′-defective and 5′-defective proviruses were 79 (IQR, 406–25), 313 (IQR, 701–44) and 126 (IQR, 247–26) per 10^6^ PBMCs, respectively (Figure 1B). Consistent with previous studies, the frequency of intact provirus was lower than that of defective proviruses, although it was not statistically significant [43,48]. In PWH with opioid use, median frequencies of intact, 3′-defective and 5′-defective proviruses were 57.62 (IQR, 512–22), 109.8 (IQR, 621–28) and 132.5 (IQR, 194–23) per 10^6^ PBMCs, respectively. Median provirus levels in the control population were comparable (intact, 203 (IQR, 406–25); 3′-defective, 392 (IQR, 701–78); 5′-defective, 92 (IQR, 287–26) per 10^6^ PBMCs). The differences between intact and defective provirus levels were not statistically significant between PWH with current opioid use and PWH without opioid use (Figure 1B). Moreover, despite having statistically significant differences in duration on ART which could potentially impact reservoir size, we observed similar levels of intact proviruses in both groups (Table 5; Figure 1B). Overall, these results suggest that opioid use has minimal impact on the shaping of the general characteristic of the proviral landscape in vivo.

### 3.2. Reactivation of HIV-1 from Ex Vivo Stimulated PBMCs Is Limited in PWH Using Opioids

**Figure 2:**
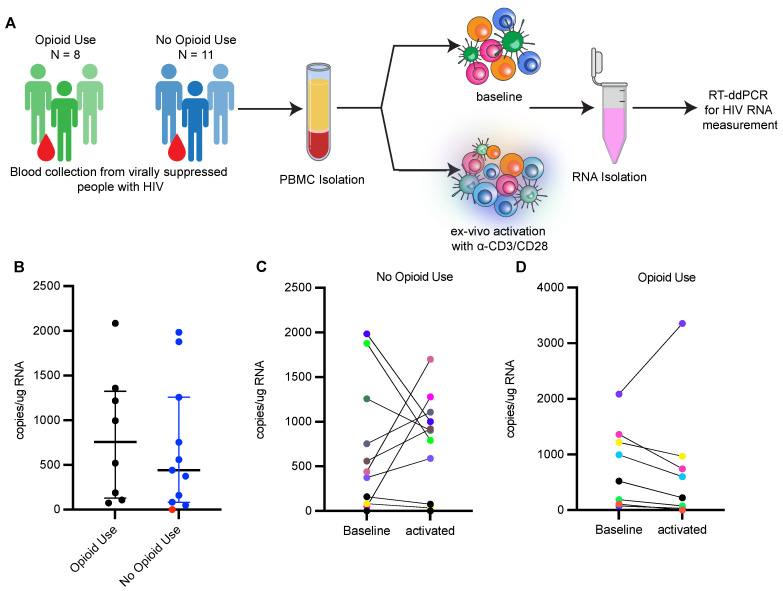
Reactivation of HIV-1 is limited in people with HIV currently using opioids. (**A**) Experimental workflow to measure HIV transcriptional reactivation following ex-vivo stimulation of PBMCs from ART suppressed PWH. (**B**) HIV transcripts detected in unstimulated PBMCs from ART treated PWH with (*n* = 8) and without current opioid use (*n* = 11). Values are represented as copies/μg RNA. Red dots indicate samples for which HIV transcripts were not detected at baseline level. Bars represent median with interquartile range. (**C**) HIV transcripts in unstimulated PBMCs and ex vivo stimulated PBMCs from ART treated PWH with current opioid use (*n* = 8). (**D**) HIV transcripts in unstimulated PBMCs and ex vivo stimulated PBMCs from ART treated PWH without current opioid use (*n* = 8). Each color represents an individual participant.

To evaluate whether opioid use impacts the induction of the latent reservoir, we measured the size of the inducible HIV-1 reservoir in PBMCs from participants enrolled in the two cohorts by activating PBMCs ex vivo with anti-CD3/CD28 beads and measuring increases in HIV-1 transcripts upon activation (Figure 2A). Primers spanning HIV-1 LTR and HIV-1 *gag* were used to detect longer, processive HIV-1 transcripts via RT-ddPCR as a measure of transcriptional elongation [49]. Net increases in HIV-1 transcripts upon ex vivo activation of PBMCs indicate reactivation of the latent reservoir. We detected HIV-1 transcripts in 18 out of 19 unstimulated baseline samples, and the transcript level was similar between the two groups (median, 757 copies/μg RNA in PWH with opioid use; 440 copies/μg RNA in PWH without opioid use) (Figure 2B). After ex vivo activation, HIV-1 transcripts were induced in 5 of the 11 clinical samples obtained from PWH without current opioid use (Figure 2C). However, only a single sample of the eight examined from PWH with current opioid use induced HIV-1 transcription upon reactivation (Figure 2D). We observed no change or decrease in HIV-1 transcripts after activation in the other seven samples, suggesting a limited HIV-1-inducible reservoir. The apparent decrease in HIV-1 transcripts after ex vivo activation may reflect an increase in total cellular RNA, which is used to normalize the HIV mRNA data. We used multiple logistic analysis (Table 6) to assess for differences in ex vivo HIV reactivation in samples from PWH with opioid use compared to PWH without opioid use, to account for differences in ART duration (Table 5; *p* = 0.0014) and levels of proviral genomes detected (Figure 1). Multiple logistic regression analysis supported that people with opioid use as compared to those without current opioid use had significantly lower odds (odds ratio (OR) 0.00951, 95% confidence interval 2.6964 × 10^−7^ to 0.1565, Table 6) of ex vivo reactivation after accounting for ART duration. Levels of intact provirus were not associated with ex vivo reactivation (Table 6). These data suggest PWH reporting current opioid use have a lower response to ex vivo reactivation as compared to PWH not currently using opioids.

### 3.3. PWH Using Opioids Have Lower Plasma TNF-α Concentration

**Figure 3:**
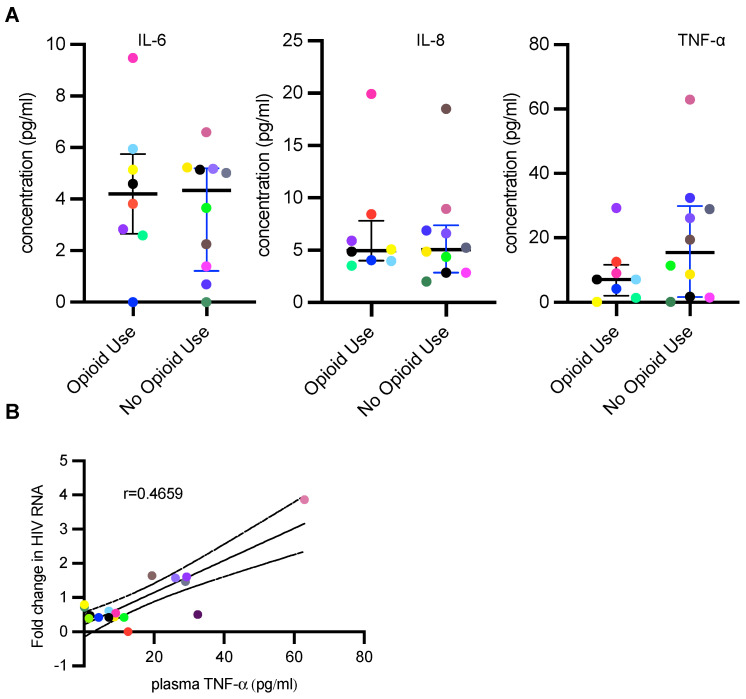
People with HIV with current opioid use disorder have lower levels of plasma TNF-α. (**A**) Measurement of IL-6, TNF-α, IL-8 concentration in frozen plasma samples from PWH with and without current opioid use. Plasma sample from one participant with no opioid use was not available. Values are represented as pg/mL. Bars represent median with interquartile range. (**B**) Spearman correlation analysis of plasma TNF-α level and fold change in HIV RNA upon activation of PBMCs ex vivo of people with current opioid use and without current opioid use, *P* value for Spearman correlation = 0.0513. Black and dotted lines represent linear regression and 95% confidence interval, respectively.

There are conflicting reports as to the impact of opioid use on inflammation. We measured IL-6, IL-8 and TNF-α, cytokines associated with inflammation, in plasma from participants from both cohorts to determine correlations between inflammatory cytokines and opioid use. A plasma sample from one participant with no opioid use was not available. There were no significant differences in IL-6 and IL-8 plasma levels between the two cohorts (Figure 3A). However, plasma TNF-α levels trended lower in PWH using opioids compared to those without current opioid use, without statistical significance (Figure 3A). Plasma TNF-α levels showed a moderate correlation with fold change in RNA upon PBMC activation ex vivo (r = 0.4659, *p* = 0.0513) (Figure 3B). It should be noted that the only participant in the PWH using opioids to show an increase in HIV-1 transcription upon ex vivo activation had elevated levels of TNF-α compared to others in this cohort. These results suggest that PWH with opioid use disorder have repressed inflammatory cytokine responses and this correlates with the inability to activate latent reservoirs.

### 3.4. Morphine Has No Impact on HIV Infection or Establishment of Latent Infection In Vitro

**Figure 4:**
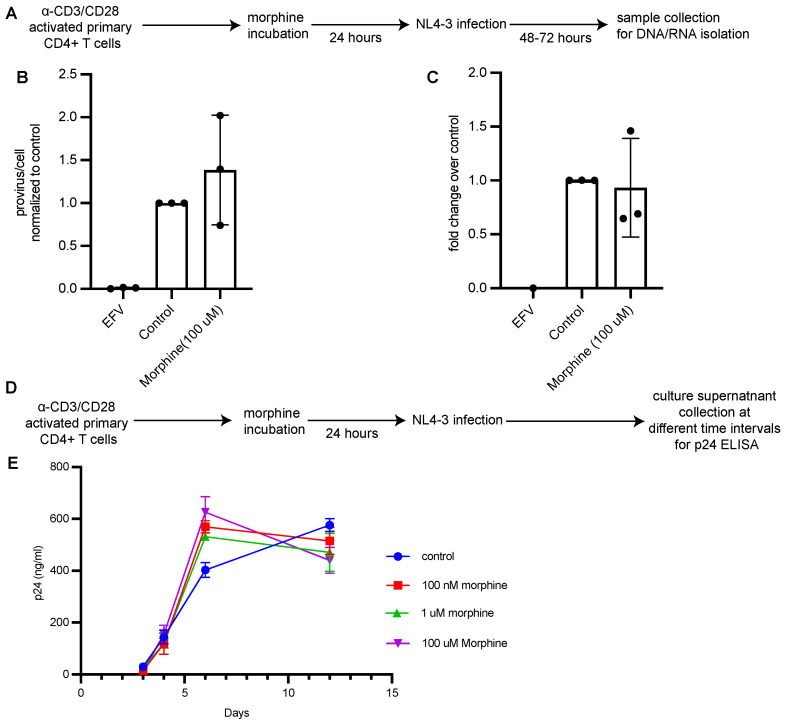
Morphine does not have an impact on HIV replication in CD4+ T cells in vitro. (**A**) Experimental workflow. Activated CD4+ T cells were pretreated with morphine (100 μM) for 24 h before in vitro infection with a single round VSV-G pseudotyped HIV-1 and cells were collected 48–72 h post infection. (**B**) Integrated HIV provirus was quantified by Alu-Gag qPCR assay. Values are represented as provirus/cell and normalized to control condition. EFV was used as an infection control to monitor plasmid contamination. Data are presented as mean and error bars represent standard deviation. *N* = 3 independent experiments from 3 donors. (**C**) HIV Tat RNA expression in cells treated with morphine was detected using RT-qPCR and was normalized to control condition. Data are presented as mean and error bars represent standard deviation. *N* = 3 independent experiments from 3 donors. (**D**) Experimental workflow. Activated CD4+ T cells were pretreated with morphine (100 uM) for 24 h before in vitro infection with a replication competent HIV-1 and cultured for 2 weeks. (**E**) Culture supernatant was analyzed for p24 levels by performing ELISA. Values are represented as ng/ml of p24 in culture supernatant and each point represents mean with standard deviation. Data are representative of 3 separate infections from one donor.

The above results suggest that PWH using opioids had a less reversible reservoir relative to the non-using group, leading us to investigate whether opioids influence HIV replication in CD4+ T cells and HIV latency reactivation directly in vitro. CD4+ T cells were enriched from PBMCs from healthy donors by negative selection using magnetic beads and were activated in vitro with anti-CD3/CD28 beads for 3 days. Activated CD4+ T cells were cultured with 100 μM morphine for 24 h, infected in vitro with a single-round VSV-G pseudotyped HIV-1 and assessed for proviral integration and transcription. We chose to use morphine because heroin, which is frequently injected by people who use opioids, is rapidly metabolized to morphine in vivo. On day 3 post-infection, cells were harvested, and DNA and RNA were isolated for qPCR analysis of integrated HIV-1 DNA and HIV RNA per cell (Figure 4A). To analyze virus replication in the presence of morphine, activated CD4+ T cells were cultured with morphine concentrations ranging from 100 nM to 100 mM for 24 h and infected with a replication-competent HIV-1. Cell culture supernatants were collected at different timepoints to assess for p24 release (Figure 4D,E). No differences in integrated HIV-1 DNA and HIV-1 RNA expression and p24 in supernatants were detected in conditions treated with morphine compared to controls, suggesting that morphine had no effect on HIV integration, transcription and replication.

**Figure 5:**
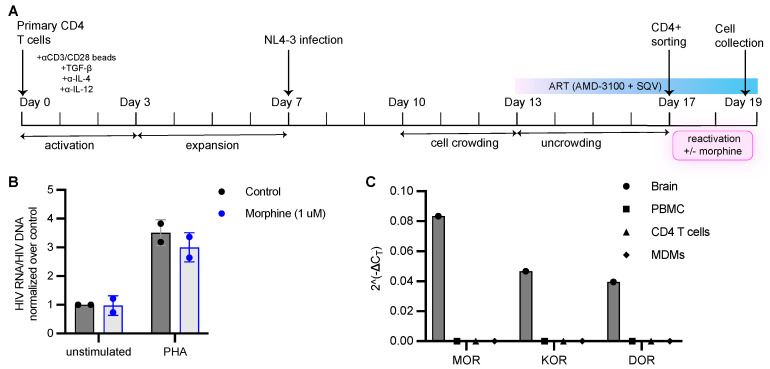
Morphine does not affect latency reactivation in vitro using a primary cell model of HIV latency. (**A**) Schematic for the generation of human primary memory T cells and subsequent establishment of latent infections. (**B**) HIV latently infected cells generated from a primary cell model of HIV latency were either left unstimulated with or without morphine or activated with PHA with or without 1 μM morphine. Following 48 h of PHA stimulation, cells were harvested for HIV DNA and RNA measurement via qPCR. HIV RNA was normalized to HIV DNA levels and fold change in HIV RNA/DNA ratio over unstimulated condition was calculated. Data are shown as mean and error bars indicate standard deviation. *N* = 2 independent experiments from 2 donors. (**C**) Opioid receptor expression in brain (inferior temporal gyrus), PBMCs, CD4+ T cells and monocyte-derived macrophages (MDMs). Y axis represents 2^(−ΔCT)^, where ΔCT = CT (opioid receptor)−CT (RPL13A). MOR represents mu opioid receptor; KOR represents kappa opioid receptor and DOR represents delta opioid receptor.

To analyze the effects of opioids on latency reactivation in vitro, we generated HIV-1 latently infected cells in vitro using a primary CD4+ T cell model of latency previously described by Bosque and Planelles (Figure 5A) [40]. Naïve CD4+ T cells were isolated from PBMCs from healthy donors and activated with anti-CD3/CD28 beads in presence of TGF-β, anti-IL-4 and anti-IL-12 antibodies to generate non-polarized, central memory-like CD4+ T cells (T_CM_) which were infected with a replication-competent NL4-3 virus. Infected T_CM_ were crowded together to facilitate viral spread for 3 days followed by the addition of antiretroviral drugs, 100 nM AMD-3100 or 1 μM of Raltegravir and 0.5 μM of Saquinavir, to prevent further viral spread for an additional 4 days. On the 17th day, CD4+ T cell enrichment was performed to remove productively infected cells from the culture based on the downregulation of CD4 in productively infected cells and high CD4 expression in uninfected and latently infected cells. This latter population was activated with PHA to ascertain the establishment of latently infected cells and confirm the reactivation of proviral transcription. Upon activation, we observed an increase in cell-associated HIV RNA indicating reactivation of latent HIV (Figure 5B). Latently infected CD4+ T cells were also cultured (without stimulation and with PHA stimulation) in the presence of morphine for 48 h, and HIV RNA copies were quantified via qPCR. We used 1 μM of morphine as we did not observe any change in activity for morphine concentrations spanning 10 nM to 1 μM (Supplementary Figure S2). We did not observe any changes in HIV transcription when latently infected cells were cultured in presence of morphine. Morphine also did not suppress PHA-induced HIV reactivation from latently infected cells (Figure 5B). These results suggest that opioids do not influence HIV expression in latently infected CD4+ T cells generated in vitro. 

The inability of morphine to influence HIV-1 replication could be explained by a lack of opioid receptor expression in immune cells. Reports that CD4+ T cells express opioid receptors have been contradictory. Therefore, we examined the expression of the classical opioid receptors, mu opioid receptor (MOR), kappa opioid receptor (KOR) and delta opioid receptor (DOR), in PBMCs, CD4+ T cells and monocyte-derived macrophages (MDMs) by RT-qPCR. Opioid receptor transcripts were not detected in any of the primary immune cells tested, whereas all three were highly expressed in inferior temporal gyrus brain tissue (Figure 5C). These data indicate that the impact of opioids on HIV latency seen in the clinical samples may not be due to a direct impact of opioids on the immune cells and reflects larger systemic effects of opioids.

## 4. Discussion

Opioid use and intravenous drug use have been linked to increased risks of HIV-1 infection and severity of disease. Although substance use disorders exacerbate several risk factors, including shared needle use, unsafe sexual practices, decreased ART adherence and loss of care continuum, it has been posited that opioids have a direct impact on CD4+ T cells and myeloid cells, influencing their ability to be infected and support HIV-1 replication. How opioids impact HIV infection, replication and the establishment and maintenance of latency is not well understood, and previous reports examining the impact of opioids on HIV-1 infection and latency using animal models and in vitro models have yielded conflicting results. In this study, we investigated the effect of opioid use on HIV reactivation from latent reservoirs in clinical samples by using PBMCs from ART-suppressed PWH that were using opioids as well as primary cell models of latency. To our best knowledge, this is the first study looking at latency reactivation in a cohort of ART-treated PWH with current opioid use. 

We examined the effects of opioid use on HIV-1 latent reservoir composition and size by analyzing PBMCs from cohorts of ART-suppressed PWH with or without opioid use at the time of study enrollment using an IPDA, which quantifies intact and defective proviruses. We generated primers and probes for HIV subtype A, which is the most common subtype in Russia, and created novel IPDA reagents as the original assay only described reagents specific for subtype B strain which accounts for 12% of global HIV infection cases as opposed to subtype A which is responsible for 24.8% of HIV infections worldwide [47,50]. Our IPDA results show that levels of both intact and defective proviruses were similar between the populations despite a statistically significant difference in duration on ART between the two groups. Our results suggesting a minimal impact of recent opioid use on the proviral landscape are consistent with a study examining the effects of heroin and cocaine use on latent reservoirs which reported no effect of either drug on intact and defective proviruses [51]. A study examining proviruses in a morphine-dependent simian immunodeficiency virus (SIV)-infected, ART-suppressed rhesus macaque model also reported no change in intact provirus frequency in PBMCs of morphine-administered monkeys compared to controls [35]. Taken together, our current results, as well as previous reports, suggest that opioids have a minimal impact on persistent proviruses. 

We extended our study by stimulating PBMCs from ART-suppressed PWH ex vivo to determine whether there were differences in the ability to induce HIV-1 expression from latently infected cells in the cohorts. We observed that despite similar proviral landscapes between PWH with and without current opioid use, the ability to reactivate HIV-1 in PWH using opioids was suppressed. Current opioid use, but not duration of ART, was predictive of suppression of latency reactivation. This suppression was moderately correlated with differences in plasma levels of TNF-α, possibly indicating that inflammation influences the establishment or maintenance of latency. Related to this, elevated TNF-α was observed in the one participant in the opioid use cohort that induced HIV-1 transcription. Diminished TNF-α levels in opioid users are consistent with previous reports showing opioids have immunosuppressive properties and reduce pro-inflammatory cytokine responses [7,52,53]. Additionally, previous single-cell RNA sequencing data from our groups support the observation that opioids have immunosuppressive properties for a range of immune cells including CD4+ T cells [54]. This observation could explain in part the association of opioid use with increased incidences of serious infections resulting in hospitalization [55]. 

To gain insight into the mechanism of action of opioids, we examined the effects of opioids on HIV-1 infection, replication and establishment and maintenance of latency using in vitro primary cell models. We did not see an effect of morphine on HIV reactivation from latently infected cells generated in vitro. Adding morphine alone did not result in the induction of HIV-1 expression from latent cells, nor did morphine influence HIV reactivation upon T cell activation via TCR engagement in vitro. These results are in agreement with reports from other groups showing morphine, heroin, buprenorphine and methadone had no effects on HIV-1 reactivation from T cell lines that harbor latent HIV [17,56]. Our data do contradict other studies reporting HIV-1 reactivation by morphine ] in the promonocytic cell line U1, which harbors repressed HIV-1, cocultured with microglia in the presence of morphine and LPS [57]. Differences in primary cells versus cell lines, mechanisms of latency and the presence of microglia-produced mediators could explain the discrepancy in our observations. Furthermore, since we did not directly treat cells with heroin, we cannot rule out the possibility it has activities that alter HIV-1 infection and latency reactivation.

Although there are several reports of immune cells expressing classical opioid receptors, mu, delta and kappa receptors [58,59,60,61], there are numerous contradictory reports about opioid receptor expression and the impact of opioids on immune function [17,62,63]. We did not detect opioid receptor mRNA in human PBMCs, CD4+ T cells or monocyte-derived macrophages, and opioids had minimal effect on HIV-1 infection and replication in vitro. However, our results do not rule out the possibility of a small percentage of immune cells expressing opioid receptors, and bulk RNA sequencing data revealed low expression of opioid receptors on some immune cells [54]. In addition, it has been reported that immune cells may express non-classical opioid receptors such as the nociceptin opioid receptor [17,54]; however, we did not examine the cells for expression of these receptors. Because of the low expression of opioid receptors by a small proportion of immune cells, we speculate that opioid-mediated regulation of HIV reactivation from PBMCs is via an indirect effect. 

Our study does have limitations. Notably, we had a limited sample size, with 19 samples in total that included 8 opioid users and 11 not using opioids diminishing our statistical power. The small sample size reflects challenges in recruiting participants and recovering viable cells from some of our clinical samples. The limited number of clinical samples did prevent analysis for confounding factors in our multivariable modeling such as polysubstance use including tobacco and alcohol which were used by individuals in both cohorts. The sample size also prevented us from accounting for HCV infection and its potential to influence HIV latency and reactivation. Importantly, our data do support a correlation between opioid use and induction of the latent reservoir. Furthermore, our study underscores the importance of using clinical samples from PWH since in vitro systems yielded very different results suggesting a minimum impact of opioids on the maintenance and reversal of latency. Another potential limitation of our study is the use of peripheral blood samples as a surrogate for latent reservoirs in different tissues which could be more sensitive to opioids, including the CNS. However, previous reports have shown significant correlations between reservoir size in blood and tissue compartment [64]. Furthermore, we used anti-CD3/CD28 antibodies to induce HIV-1 expression. It would be valuable to examine how opioid use influences HIV-1 induction by different classes of latency-reversing agents. 

The discrepancy in our data from in vitro and clinical samples suggests that opioids do not impact HIV expression in CD4+ T cells directly and highlights the importance of studying clinical samples to analyze the impact of long-term opioid use on HIV infection. Taken together, our results from clinical samples and in vitro experiments support a model in which opioids cause a systemic inhibition of immune function which indirectly impacts the maintenance of the latent reservoir in ART-suppressed PWH. 

One implication of our results is that efforts to reduce the reservoir by strategies such as “shock and kill” approaches may be less efficacious in PWH that use opioids. Furthermore, opioid-mediated immune modulation may promote a deep-seated latency that might be more difficult to reverse. For PWH who use opioids, different strategies to target that latent reservoir may be required. 

## Figures and Tables

**Table 1:** Sequences of primers and probes used for Alu-PCR.

Assay	Primer Name	Sequence (5′ → 3′)
Alu-PCR	Albumin F	GCTGTCATCTCTTGTGGGCTGT
	Albumin R	AAACTCATGGGAGCTGCTGGTT
	Albumin probe	CCTGTCATGCCCACACAAATCTCTCC
	Alu F	GCCTCCCAAACTGCTGGGATTACA
	Gag R	GCTCTCGCACCCATCTCTCTC
	HIV-1 ‘R’ F	GCCTCAATAAAGCTTGCCTTGA
	HIV-1 ‘U5’ R	TCCACACTGACTAAAAGGGTCTGA
	RU5 probe	CCAGAGTCACACAACAGACG

**Table 2:** Primers and probes used in RT-qPCR.

Assay	Target Gene	Primer Name	Sequence (5′ → 3′)
RT-qPCR	HIV Tat	Spliced Tat F	TCCCTCAGACCCTTTTAGTCAG
		Spliced Tat R	CATCTGTCCTCTGTCAGTTTC
	Mu-opioid receptor	OPRM F	TCTACTCCATCGTGTGCGTG
		OPRM R	CAGTCTTCATCTTGGTGTATCTGAC
	Delta-opioid receptor	OPRD F	CGGCATCGTCCGGTACACTA
		OPRD R	CTTGGCACTCTGGAAAGGCA
	Kappa-opioid receptor	OPRK F	CAGGTGATGCCAAGAGCTGA
		OPRK R	CCTGCGGCGCTATGGTT
	RPL13a	RPL13a F	CAAGCGGATGAACACCAAC
		RPL13a R	CGCTTTTTCTTGTCGTAGGGG

**Table 3:** Primers and probes used in intact proviral DNA assay (IPDA).

Assay	Target Gene	Primer Name	Sequence (5′ → 3′)
Intact Proviral DNA Assay	HIV Psi	Ψ F	CAGGACTCGGCTTGCTGAGG
		Ψ R	GCACCCATCTCTCTCCTTCTAGC
		Ψ Probe	ATGGCGTACTCACCAGT
	HIV Env	Env F	AGAGAGAAAAAAGAGCAGT
		Env R	GGCCTGTACCGTCAG
		Env intact probe	AGCAGGAAGCACTATGGG
	RPP30	RPP30-1 F	GATTTGGACCTGCGAGC
		RPP30-1 R	GCGGCTGTCTCCACAAG
		RPP30-1 probe	CTGACCTGAAGGCTCT
		RPP30-2 F	GACACAATGTTTGGTACATGGTTAA
		RPP30-2 R	CTTTGCTTTGTATGTTGGCAGAAA
		RPP30-2 probe	CCATCTCACCAATCATTCTCCTTCCTTC

**Table 4:** Primers and probes used for RT-ddPCR.

Assay	Primer Name	Sequence (5′ → 3′)
RT-ddPCR	R-U5 F	GCCTCAATAAAGCTTGCCTTGA -
	Gag R	GGGCGCCACTGCTAGAGA
	R-U5/Gag probe	CCAGAGTCACACAACAGACGGGCACA

**Table 5:** Descriptive characteristics of study participants.

	People with Current Opioid Use *n* = 8	People without Current Opioid Use *n* = 11
Age Mean (SD) Median (IQR)	37.8 (2.7) 38 (4)	39.18 (10.78) 37 (4.5)
Gender Male (*n*, %) Female (*n*, %)	5 (62.5%) 3 (37.5%)	7 (63.63%) 4 (36.37%)
Education ≥9 grades (*n*, %)	8 (100.0%)	11 (100.0%)
CD4+ T cell count Mean (SD) Median (IQR)	558 (312) 502 (393)	(information available for *n* = 8) 453 (148.6) 481.5 (145.5)
Time since first HIV diagnosis Mean (SD) Median (IQR)	13.3 (6.0) 12.4 (7.5)	10.52 (5.78) 10.2 (10.4)
**Current ART** Yes (*n*, %) No (*n*, %)	8 (100.0%) 0 (0.0%)	11 (100.0%) 0 (0.0%)
Time since ART initiation, yrs ^a^ Mean (SD) Median (IQR)	0.6 (0.3) 0.6 (0.4)	4.36 (2.85) 4.1 (2.95)
**HIV viral load**	<LOD *	<LOD **
**Opioid use at the time of enrollment, (*n*, %)**	8 (100.0%)	0 (0.0%)
**Other substance use (*n*, %)**		
Alcohol (AUDIT score)		
Alcohol abstainer/low-risk consumption	5 (62.5%)	9 (81.81%)
Hazardous drinking	1 (12.5%)	1 (9.09%)
Harmful drinking	0 (0.0%)	0 (0.0%)
Likelihood of alcohol dependence	2 (25.0%)	1 (9.09%)
Current tobacco smokers (*n*, %)	8 (100.0%)	6 (54.54%)
Current cannabis, past 30 days	0 (0.0%)	0 (0.0%)

^a^*p* value = 0.0014; unpaired *t*-test with Welch’s correction. * limit of detection = 50 copies/mL. ** limit of detection = 300 copies/mL.

**Table 6:** Predictors of latency reactivation in PBMCs in multiple logistic regression analysis.

Predictor (Variables)	Odds Ratio (95% CI)	*p* Value
Intercept	138.3 (1.593 to 2,598,796)	0.1322
Duration on ART (year)	0.2478 (0.01881 to 0.7682)	0.1051
Current opioid use	0.00951 (2.6964 × 10^−7^ to 0.1565)	0.0395
Intact provirus per 10^6^ PBMCs	1.00 (0.9972 to 1.003)	0.7442

Abbreviations: ART, antiretroviral therapy; CI, confidence interval.

## Data Availability

All research data supporting these results are included in the manuscript.

## Supplementary Materials

The following supporting information can be downloaded at: https://www.mdpi.com/article/10.3390/v15020415/s1, Table S1: Descriptive characteristics of study participants without ART treatment (IMPACT cohort). Figure S1: Detecting HIV subtype A by IPDA, Figure S2: In Vitro reactivation of latent HIV in the presence of different morphine concentrations.
