# Supplementary material for: Virally Suppressed People Living with HIV Who Use Opioids Have Diminished Latency Reversal
Source: Viruses. 2023 Feb 1;15(2):415. doi: 10.3390/v15020415 (PMC9961149; doi:10.3390/v15020415)
Supplement: Supplementary file 1 [file viruses-15-00415-s001.zip › viruses-2145071-table S1.pdf]

Supplementary Table S1: Descriptive characteristics of study participants without ART treatment (IMPACT cohort)

	Current Opioid users<br>N = 15	Current Opioid non-users<br>N = 20
Gender<br>Male (n, %)<br>Female (n, %)	 7 (46.7%)<br>8 (53.3%)	 13 (65%)<br>7 (35%)
Education<br>>9 grades (n, %)	 12 (80%)	 18 (90%)
CD4 cell count<br>Mean (sd)<br>Median (IQR)	 589.9 (323.7)<br>559 (386)	 596.9 (327.1)<br>584 (474)
HIV viral load at blood draw (total copies/ml)<br>Median (IQR)	 20500 (63896)	 102369 (84869)
Time since first HIV diagnosis<br>Mean (sd)<br>Median (IQR)	 5.4 (3.2)<br>4.8 (5.9)	 6.1 (3.5)<br>4.8 (6.5)
**Current ART**		
Yes (n, %)	0 (0%)	0 (0%)
No (n, %)	15 (100%)	20 (100%)
**Opioid use at the time of enrollment (n,%)**	15 (100%)	0 (0%)
**Other substance use (n, %)**		
Risky alcohol drinking (NIAA criteria)<br>n, %	 9 (60%)	 8 (40%)
Tobacco (n, %)	15 (100%)	16 (80%)
Current Cannabis, past 30 days<br>n, %	 6 (40%)	 2 (10%)
